# Use of Acellular Dermal Matrix to Prevent Recurrence of Radioulnar Heterotopic Ossification

**DOI:** 10.1097/GOX.0000000000002257

**Published:** 2019-06-14

**Authors:** Daniel J. Gould, Paymon Rahgozar, Eric S. Nagengast, David A. Kulber

**Affiliations:** From the * Division of Plastic and Reconstructive Surgery, University of Southern California Keck School of Medicine, Los Angeles, Calif.; † Division of Plastic and Reconstructive Surgery, University of California San Francisco, San Francisco, Calif.; ‡ Department of Orthopedic Surgery, Cedars-Sinai Medical Center, Los Angeles, Calif.; § Department of Surgery, Cedars-Sinai Medical Center, Los Angeles, Calif.

## Abstract

Radioulnar heterotopic ossification is a rare occurrence found in approximately 2% of all forearm injuries. Treatment is complicated by relatively high recurrence rates. Strategies to decrease recurrence have included the range of motion exercises and the interposition of inert or autogenous barriers. We report on the interposition of human acellular dermal matrix (ADM) for the treatment of distal radioulnar synostosis. We report a novel technique for the treatment of distal radioulnar heterotopic ossification. After resection, ADM in a cigar-shaped construct is interposed between the radius and ulna. Patients are followed clinically and radiographically. Two female patients were treated. Both patients had significant improvement in the range of motion in supination and pronation of the affected wrist postoperatively with an average follow-up of 36 months. There were no postoperative complications. Neither patient had recurrent disease. We describe the successful treatment of 2 patients with distal radioulnar heterotopic ossification with the use of human ADM. The ADM provides a barrier between the radius and ulna to prevent the recurrent formation of heterotopic ossification. ADM usage results in no donor site morbidity and is theoretically more resistant to infection when compared with nonbiologic barriers such as silicone and Integra. This technique is a simple, safe, and effective way to treat and prevent the recurrence of radioulnar heterotopic ossification.

Radioulnar synostosis due to heterotopic ossification is well described with an estimated occurrence in 2% of forearm injuries.^1^ The primary consequence is the impairment of forearm pronation and supination. Risk factors include fracture of both forearm bones at the same level, open fracture, significant soft tissue injury, high-energy fracture, cranial trauma, spinal cord injury, and bone fragments on the interosseous membrane.^2,3^

Treatment requires direct excision of the heterotopic ossification. However, recurrence after surgical resection remains a significant problem with an estimated average recurrence rate of 32%.^4^ Strategies to decrease recurrence have included the range of motion exercises and the interposition of inert or autogenous barriers such as silicone, dermal/silicone sheet implant, bone wax, free fat, vascularized muscle interposition, vascularized free flaps, and vascularized pedicle flaps.^1,4–11^ Furthermore, investigation of adjuvant treatments including nonsteroidal anti-inflammatory medications (NSAIDs) and low-dose postoperative irradiation has shown mixed results.^10,11^ Many of the methods to reduce recurrence have significant morbidity including but not limited to donor site morbidity, need for a second operation, increased risk of bleeding, or wound healing problems.

Used in multiple surgical specialties including hand surgery, human acellular dermal matrix (ADM) is sterilized decellularized human dermis that retains elastin and collagen fibers and has the ability to act as a framework to support cellular repopulation and vascularization.^12^ A significant advantage of ADM is no donor site morbidity. Here, we describe a technique using the interposition of ADM to successfully treat and prevent recurrent radioulnar synostosis.

## CASE REPORT

### Case 1

**Fig. 1.**
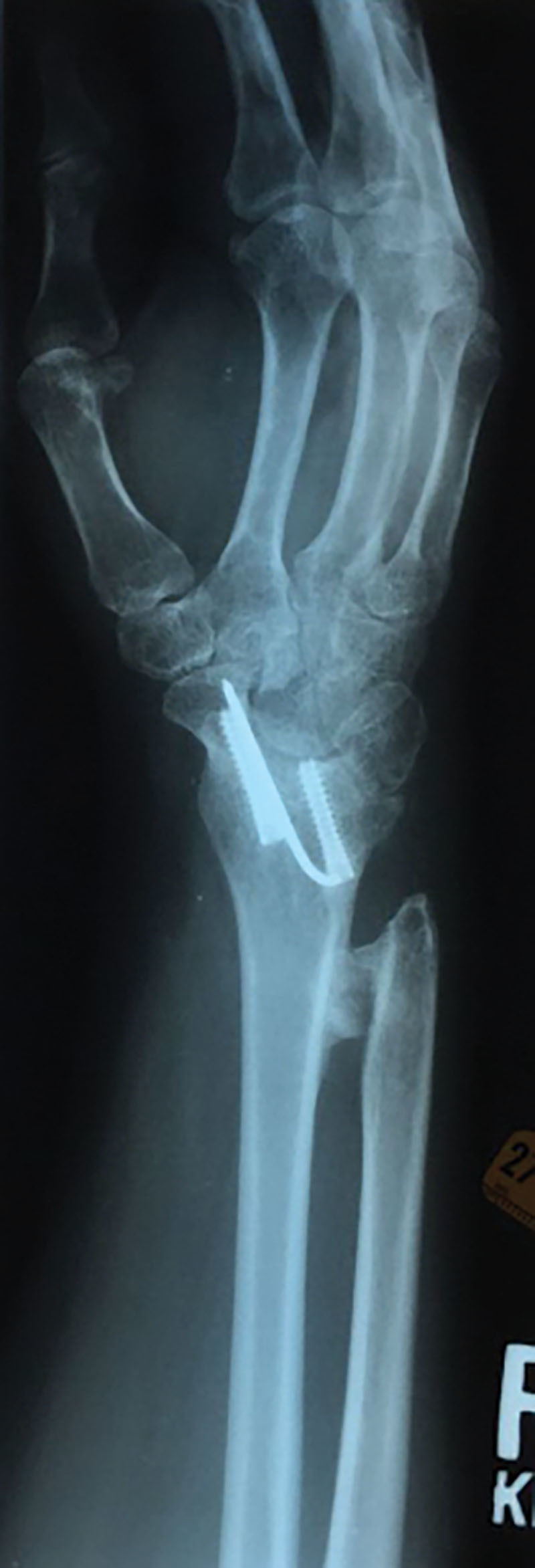
Patient 1 preoperative roentgenogram showing heterotopic ossification of the right distal radius and ulna.

The first patient is a 54-year-old woman with a history of arthritis, fibromyalgia, and hypothyroidism who had initially undergone an arthroscopic debridement of a right triangular fibrocartilage complex and scapholunate ligament tear. The patient had persistence of symptoms and decreased range of motion. She received multiple steroid injections to the radiocarpal joint without relief. Radiographs showed progressive radiocarpal arthritis, and she elected to proceed with a radiocarpal fusion with a Darrach resection 15 months later. While the patient’s pain improved, she complained of decreased range of motion in the wrist. On examination, she had 0 degrees of pronation and supination of the right wrist. Plain radiographs confirmed synostosis of the interosseous membrane of the distal radius and ulna secondary to heterotopic ossification (Fig. 1).

She was taken to the operating room, and after axillary block anesthesia, the extremity was exsanguinated and a tourniquet inflated. A longitudinal incision was made along the ulnar side of the dorsum of the distal extremity. The extensor carpi ulnaris tendon and the ulnar sensory nerve were identified and retracted. The slips of the extensor digitorum communis tendons were elevated from the soft tissue, exposing the heterotopic ossification. With intraoperative fluoroscopy, the heterotopic bone was completely resected with the aid of a sagittal saw, osteotome, and rongeur. Afterward, the forearm could be supinated and pronated without difficulty.

**Fig. 2.**
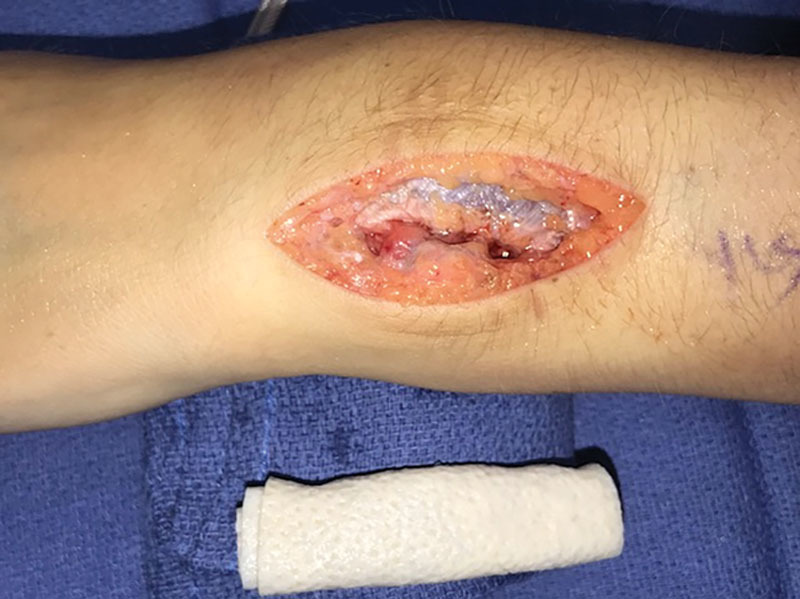
ADM construct prepared in a cigar-like fashion before implantation.

**Fig. 3.**
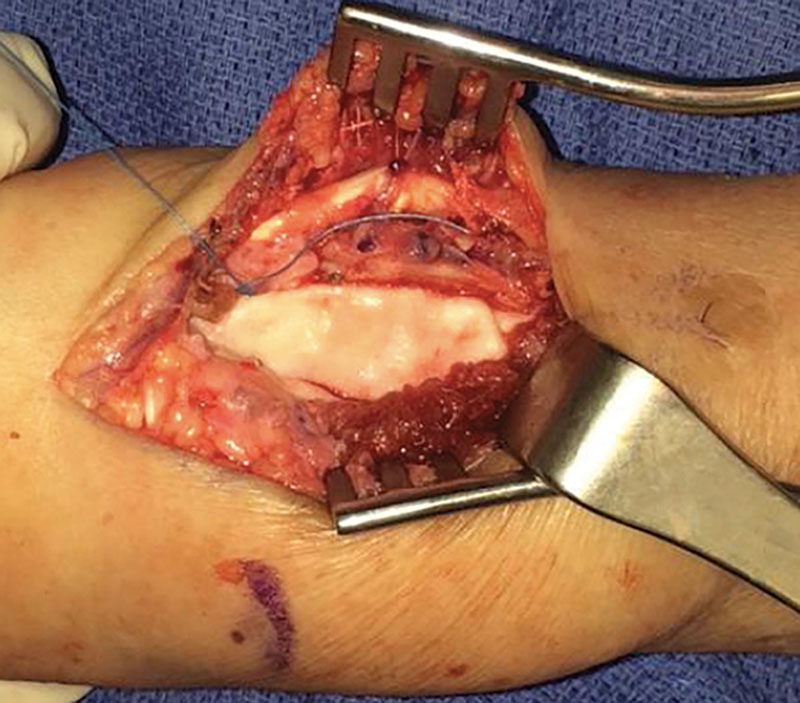
ADM construct interposed between the radius and ulna after resection of the heterotopic ossification.

A 8 mm × 12 mm × 0.5 mm piece of FlexHD ADM (FlexHD Structural; Musculoskeletal Transplant Foundation, Edison, NJ) was prepared in a cigar fashion with the dermis facing outward, by suturing one edge to the other using 3–0 Tycron sutures (Fig. 2). The ADM construct was secured to the base of the radioulnar joint volar to the pronator teres muscle with TiCron (Medline, Northfield, IL) sutures placed proximally and distally. Additionally, Mersilene (Ethicon US, LLC., Bridgewater, NJ) sutures were placed to secure the ADM to the ulna and radius after suture holes were created with a k-wire (Fig. 3). Care is taken to completely cover the area previously spanned by the heterotopic ossification. Supination and pronation were tested again, and the ADM was inspected confirming stable positioning. The soft tissues were closed in layers, and capillary refill was verified after the deflation of the tourniquet. The patient was placed in a volar splint for 3 weeks. She then began therapy including strengthening, flexibility, and functional retraining.

**Fig. 4.**
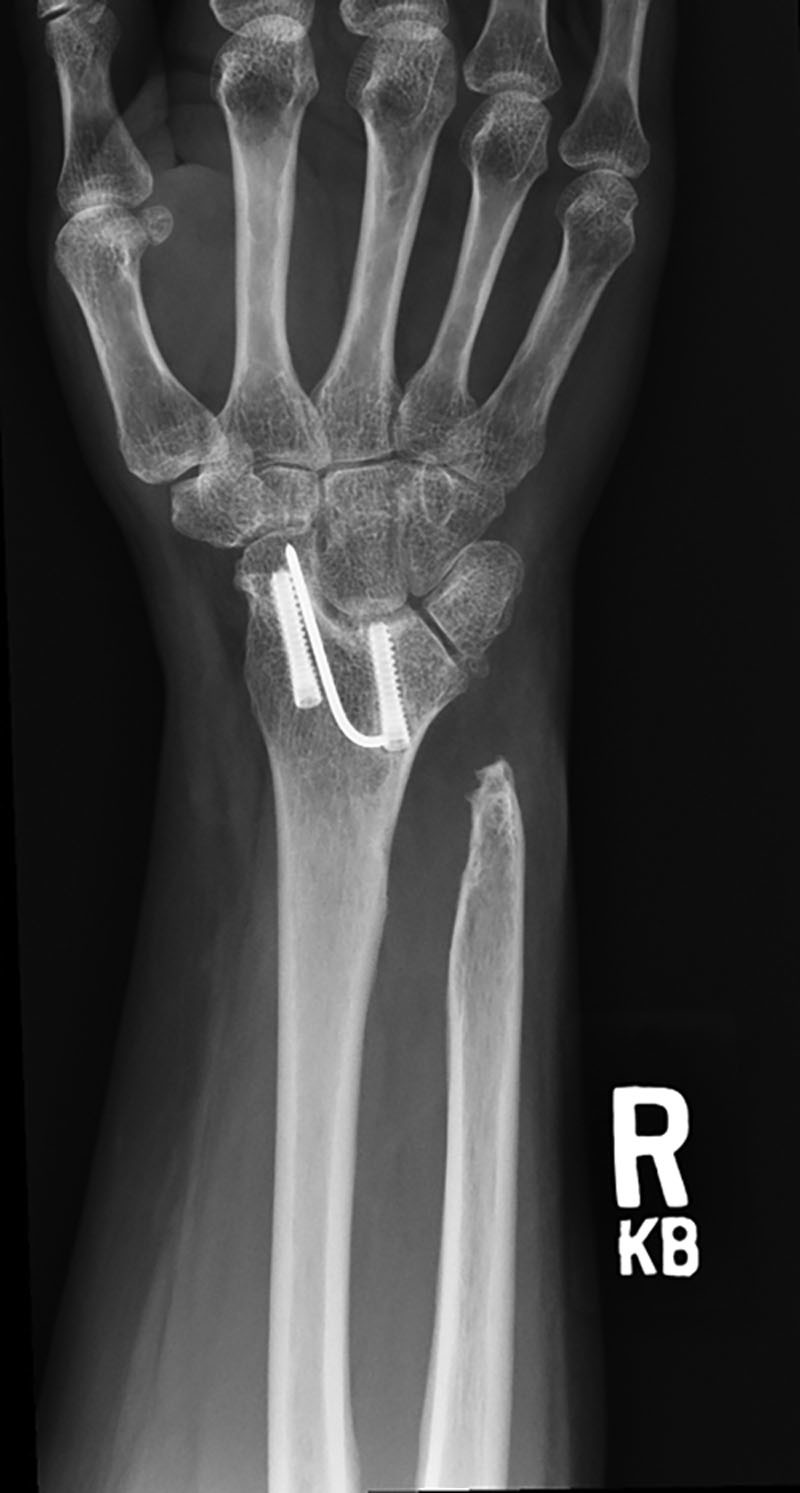
Patient 1 9-month postoperative roentgenogram showing no recurrence of heterotopic ossification.

At 39 months follow-up, the patient has significant improvement in the range of motion with 50 degrees pronation and 70 degrees supination and no radiographic evidence of recurrent distal radioulnar synostosis (Fig. 4).

### Case 2

**Fig. 5.**
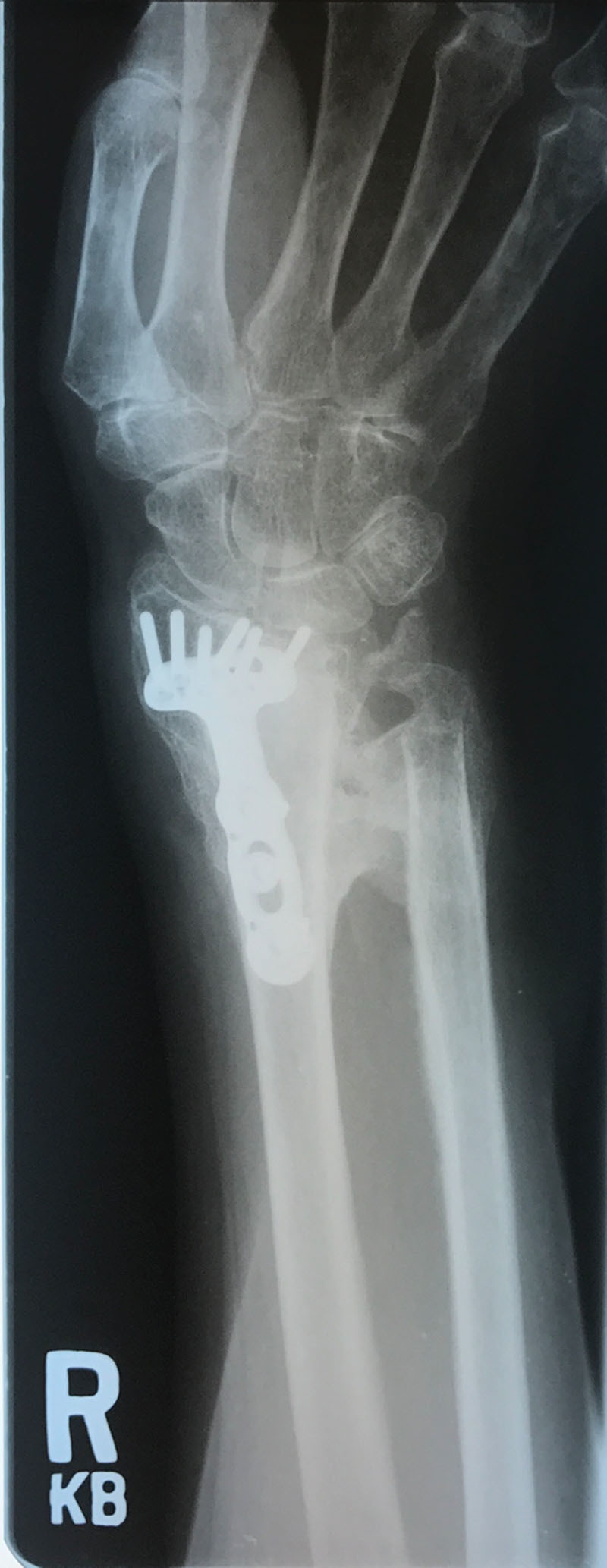
Patient 2 preoperative roentgenogram showing heterotopic ossification of the right distal radius and ulna.

**Fig. 6.**
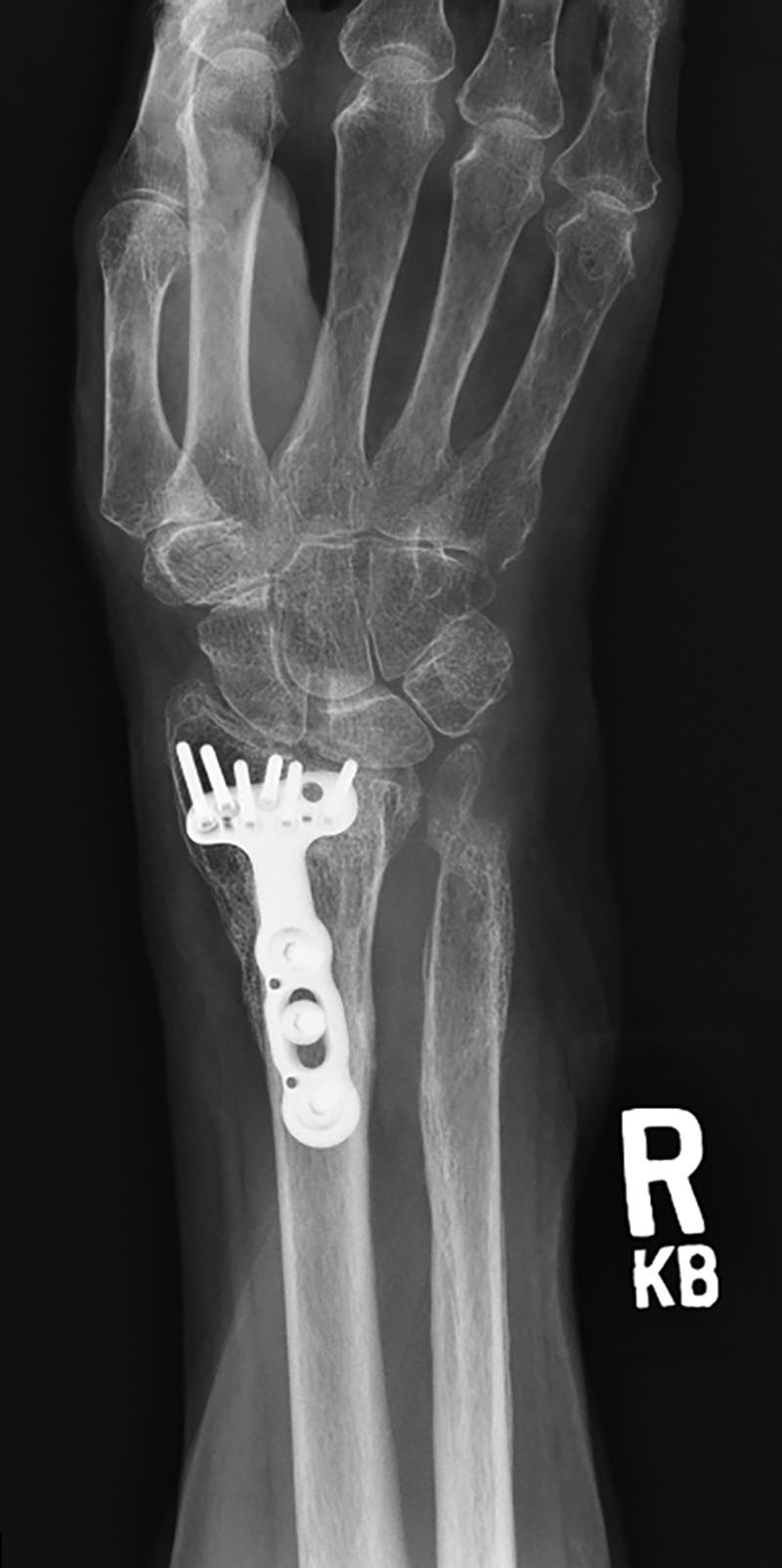
Patient 2 8-month postoperative roentgenogram showing no recurrence of heterotopic ossification.

The second patient is an otherwise healthy 83-year-old woman with a history of a ground level fall resulting in a severely comminuted, intra-articular, right, distal radius fracture with a displaced ulna fracture. She had an open reduction with internal fixation of the distal radius with a Darrach resection and repair of the dorsal capsule. At 6 months, the fracture was well healed, but the patient was unable to pronate or supinate the arm, and radiographs confirmed heterotopic ossification between the radius and ulna (Fig. 5). The patient had surgical resection of the heterotopic ossification and placement of ADM as described earlier with intraoperative restoration of 60 degrees of pronation and 60 degrees of supination. At 33 months, she continues to have a 120-degree total arc of pronation/supination and no radiographic evidence of recurrent synostosis, though there is evidence of heterotopic ossification at the distal ulna (Fig. 6).

## DISCUSSION

Radioulnar heterotopic ossification is a well-described phenomenon that can result in ankylosis, pain, and decreased ability to pronate/supinate the forearm. The treatment is primarily surgical, complicated by recurrence rates ranging from 5% to 60%.^4^ Vince and Miller,^1^ who devised the classification scheme of radioulnar cross-union showed that distal intra-articular radioulnar synostosis has a high rate of recurrence occurring in 3 of the 4 patients in their review. Multiple methods to prevent recurrence are described, but there is no consensus as to which method is best.

Nonsurgical techniques are described to prevent heterotopic ossification including the use of NSAIDs and postoperative irradiation. Most of these data, however, come from studies following hip arthroplasty.^13–15^ Jupiter and Ring^10^ evaluated a consecutive series of 18 limbs noting no significant difference in the rate of recurrence of post-traumatic proximal radioulnar synostosis with the use of autologous free fat graft, the lack of postoperative NSAIDs, or radiation. Their only recurrence was a case involving initial cranial trauma. For radioulnar synostosis, these medical treatments are often used in combination with surgery.

Though no comparative study is large enough to show significance, the existing data suggest that providing an effective barrier between the radius and ulna helps prevent the recurrence of heterotopic ossification. Multiple studies use autogenous tissue interposition for the treatment of heterotopic ossification. Fernandez and Joneschild^4^ successfully used a proximally based pedicled brachioradialis flap to treat recurrent radioulnar synostosis in 5 patients with no recurrence over a mean of 8 years. Sonderegger et al^11^ described the use of a pedicled adipofascial flap based on either the radial artery or the posterior interosseous artery in 6 patients, five patients with proximal radioulnar synostosis and one patient with distal radioulnar synostosis. At a mean follow-up of 32 months, all patients had good pronation and supination with no radiologic recurrence. There was one complication of a transient posterior interosseous nerve palsy. Harvesting of autogenous tissue increases operating time and the potential for donor site complications. Friedrich et al^8^ reviewed 13 patients with radioulnar heterotopic ossification treated with tensor fascia lata interposition after synostosis resection with good results. Two of their 13 patients had strictly distal disease. There was one complication of a wound dehiscence but no cases of recurrence. Interestingly, the first 3 patients in their series had autologous fascia lata grafts, but the subsequent 10 patients had a cadaveric source to avoid such a large donor site.^8^ In this technique, the authors wrapped one of the bones with the graft, requiring more dissection than the technique described in this article. Both treatments are similar in that they have no donor site, and both could be considered.

Although autogenous tissue has promising results in a limited number of studies, Failla et al^3^ in their series of post-traumatic proximal radioulnar synostosis observed superior outcomes in patients treated with the interposition of nonbiologic material (silicone) as compared with a biologic substance such as muscle, fascia, and fat. The proposed reason for this is an increase in scaring caused by the autogenous tissue. Similar to silicone, Lytle and Chung^9^ described a case of a distal radioulnar synostosis treated with Integra dermal regeneration template (Integra Bilayer Matrix Wound Dressing; Integra Life Sciences, Plainsboro, NJ) and postoperative NSAID use with a successful result at 1 year.

ADM is a nonautologous biologic material formed from the decellularization of human dermal matrix by a controlled sterilization process that removes the epidermis and the cells from the dermis leaving dermal matrix components including collagen fibers, elastin, proteoglycans, and the vascular scaffold intact. ADM is safely used for various methods of hand reconstruction.^12^ ADM is known to neovascularize when in the human body.^16^ This property makes ADM more resistant to infection making ADM recommended for use in the repair of contaminated abdominal hernias.^17^

Though both patients had successful outcomes, this study has limitations. With only 2 patients, the study is underpowered to draw statistical conclusions. It is also difficult to determine the complications of this operation. The FlexHD used in these patients has a cost of approximately $1,600 adding a significant cost to the operation. More patients will have to be treated with this intervention and followed long-term to draw further conclusions and determine if the cost of ADM is warranted for this disease treatment.

In this report, we describe the successful treatment of 2 patients with distal radioulnar heterotopic ossification with the use of FlexHD ADM. The ADM provides a barrier between the radius and ulna to prevent the recurrent formation of heterotopic ossification with no donor site morbidity. ADM is theoretically more resistant to infection when compared with nonbiologic barriers such as silicone and Integra. The use of ADM is a simple, safe, and effective way to treat and prevent the recurrence of radioulnar heterotopic ossification.
